# The role of PAR2 in regulating MIF release in house dust mite-induced atopic dermatitis

**DOI:** 10.3389/fimmu.2024.1478292

**Published:** 2024-10-02

**Authors:** Lingxuan Zhou, Guohong Zhang, Kai Zhang, Ziyan Rao, Zhanli Tang, Yang Wang, Jiahui Zhao

**Affiliations:** ^1^ Department of Dermatology, Peking University First Hospital, Beijing Key Laboratory of Molecular Diagnosis on Dermatoses, National Clinical Research Center for Skin and Immune Disease, National Medical Products Administration (NMPA) Key Laboratory for Quality Control and Evaluation of Cosmetics, Beijing, China; ^2^ Department of Biomedical Informatics, School of Basic Medical Sciences, State Key Laboratory of Vascular Homeostasis and Remodeling, Peking University, Beijing, China; ^3^ Department of Dermatology, Qilu Hospital of Shandong University, Jinan, China; ^4^ Chinese Institute for Brain Research (CIBR), Beijing, China

**Keywords:** atopic dermatitis, house dust mite, PAR2, MIF release, single-cell transcriptomics

## Abstract

Atopic dermatitis (AD) is a chronic disease characterized by relapsed eczema and intractable itch, and is often triggered by house dust mites (HDM). PAR2 is a G-protein coupled receptor on keratinocytes and may be activated by HDM to affect AD processes. We first established a HDM-derived AD mouse model in wild-type (WT) and *Par2^-/-^* mice. Single cell RNA sequencing of the diseased skins found a stronger cellular communication between the ligand macrophage migration inhibitory factor (MIF) from keratinocytes and its receptors on antigen-presenting cells, suggesting the critical role of MIF in AD. HDM-WT mice showed severer skin lesions and pathological changes with stronger immunofluorescence MIF signals in skin sections than HDM-*Par2^-/-^* mice. Primary keratinocytes from WT mice stimulated with HDM or SLIGRL (PAR2 agonist) secreted more MIF in cultured medium and induced stronger immunofluorescence MIF signals than those from *Par2^-/-^* mice. The skin section of HDM-WT mice showed higher immunofluorescence signals of P115 (relating to MIF secretion) and KIF13B (possibly relating to intracellular trafficking of MIF) than that of HDM-*Par2^-/-^* mice. Acetylation of α-tubulin increased after stimulation by SLIGRL in WT keratinocytes but not in *Par2^-/-^* keratinocytes. HDM-WT mice treated with the MIF antagonist ISO-1 displayed improvement of AD-like presentations and lower expressions of IL-4, IL-13, TSLP and Arg1 (a biomarker of M2 macrophage) mRNAs. We conclude that MIF is an important cytokine and is significantly increased in the AD model. PAR2 affects AD changes by regulating the expression, intracellular trafficking, and secretion of MIF in epidermis.

## 1 Introduction

Atopic dermatitis (AD) is a recurrent, pruritic, inflammatory skin disease as well as the earliest manifestation of atopic march. Many patients with atopic dermatitis have increased reactivity to aeroallergens and food allergens. House dust mites (HDM), the most common environmental allergens, are considered to be important in the initiation and exacerbation of AD (1). AD patients have higher HDM-specific IgE levels, and HDM desensitization can improve the AD clinical symptoms effectively (2). Furthermore, HDM stimulates keratinocytes to release Th2-associated cytokines such as interleukin-(IL-)25 and IL-33 in AD to aggravate the immune reactions (3).

Protease activated receptor 2 (PAR2), a seven-transmembrane G protein-coupled receptor, is vital in developing AD. PAR2 hyperactivation triggers AD onset or worsens its symptoms (4). Mite proteases impact epithelial cells by causing barrier dysfunction, skin itching, and cytokine release. HDM also directly mediates the neuroimmune response in AD rather than compromising the skin barrier (5). HDM-derived serine protease cleaves an N-terminal peptide of PAR2, inducing Ca^2+^ mobilization through PAR2 activation (6). In asthma models, HDM-activated PAR2 increases expression of IL-33 and thymic stromal lymphopoietin (TSLP) from bronchial epithelial cells (7). However, the disruptions of the AD immune microenvironment following PAR2 activation by HDM remain poorly understood.

Macrophage migration inhibitory factor (MIF) is a multifunctional protein crucial in inflammatory and autoimmune diseases. MIF is involved in delayed hypersensitivity reactions, with its expression correlating with hypersensitivity severity, marking its role as a pro-inflammatory factor in allergic disorders (8). Inhibiting MIF in HDM-induced asthma models significantly reduces inflammation and airway hyper-responsiveness (9). Elevated MIF levels are observed in the serum and skin lesions of AD patients, making it an AD severity indicator (10, 11). MIF is secreted as a cytokine and can also be encapsulated in exosomes for delivery within the body. PAR2 activation increases the extracellular release of MIF and its carrier, Golgi vesicle transporter protein P115, through a non-inflammatory mechanism (12). However, the precise mechanism of PAR2-mediated MIF release in keratinocytes is unknown.

In this work, we established a novel HDM-induced AD mice model and demonstrated that PAR2 affected MIF release by regulating the binding of KIF13B to the MIF-P115 transport complex in keratinocytes, which influences cellular communication between epidermal cells and MIF-related antigen-presenting cells.

## 2 Methods

### 2.1 Mice and model

Adult male (8-10 weeks) *Par2^-/-^* and wild type C57BL/6J mice were used in this study (*Par2^-/-^* mice were purchased from Jackson Laboratory). The mice were housed at 23°C with a 12:12 light/dark circle and 50 ± 10% humidity under specific pathogen-free conditions with food and water available *ad libitum*. All experimental procedures were approval by the Animal Studies Committee at Peking University First Hospital.

To induce the HDM-allergic AD mice model, calcipotriol (MC903; 6 nmol/60 μL; Tocris Bioscience, UK) dissolved in ethanol was topically applied to a shaved area on mouse neck (2.5 cm × 2.5 cm) daily for seven days. Then, 100 mg of HDM ointment (Biostir AD, Japan) was topically applied to the same area, followed by MC903 application three times weekly. After six HDM treatments, the mice were euthanized, and back skin specimens from the treated areas were collected for analysis.

Scratching behavior was observed for 60 minutes at 5-minute intervals, as described previously (13). The AD severity index (clinical score) assessed the intensity of skin symptoms, redness, bleeding, eruptions, and scaling, scored as follows: 0, none; 1, mild; 2, moderate; 3, severe. This index was evaluated weekly throughout the experiment.

### 2.2 Single cell RNA-seq and analysis

Freshly collected skin tissues from mice were placed in MACS^®^ Tissue Storage Solution (Miltenyi Biotec, Germany). Tissue samples were digested using the Tissue Digestion Kit (Miltenyi Biotec) according to the instructions. Briefly, tissues were cut up and placed in a mixed digestive enzyme solution and incubated at 37°C for 1.5 hours. Single cell suspensions were obtained after filtration using SmartStrainer filters (Miltenyi Biotec). ScRNA-seq was conducted using the 10× Genomics Chromium platform. Raw expression data were processed with the Seurat (v3.1.5) toolkit in R (v4.0.0) for quality control and downstream analysis. Unsupervised clustering was performed, followed by visualization using UMAP. The Seurat ‘findmarker’ function identified differentially expressed genes for each cluster. Potential interactions between cell types were identified using the CellChat function.

### 2.3 Cell culture

Primary keratinocyte cultures were prepared as previously described (13). Briefly, the back skin of newborn mouse pups (P0-P3) was digested with 0.25% Dispase II (Roche, Switzerland) at 4°C overnight. The epidermis was then separated to obtain a single-cell suspension. Keratinocytes were cultured in CnT-07 medium (Advanced Cell Systems, Switzerland) with 1% Penicillin-Streptomycin (Gibco, USA) at 37°C and 5% CO_2_.

### 2.4 Immunofluorescence

Mice back skin tissues or primary keratinocytes were incubated with primary antibodies: anti-MIF antibody (1:100, Cell Signaling Technology), anti-P115 antibody (1:300, Proteintech), or anti-KIF13B antibody (1:100, Invitrogen) at 4°C overnight. Slides were then incubated with secondary antibodies: Alexa Fluor488 goat anti-rabbit IgG (1:200, Invitrogen) or Alexa Fluor594 goat anti-mouse IgG (1:200, Invitrogen) for 2 hours at room temperature. Sections were counterstained with DAPI and visualized using a confocal microscope (Leica).

### 2.5 Real-time quantitative PCR

Total RNA was extracted from mouse back skin tissues using TRIzol reagent (Invitrogen) according to standard protocol followed by reverse transcription to generate cDNA by TransScript IV One-Step gDNA Removal and cDNA Synthesis SuperMix (TransGen Biotech). Real-time PCR was performed by using Power SYBR Green PCR Master Mix (Applied Biosystems, UK) according to the manufacturer’s instructions. The primers used are listed in **Supplementary Table S1.**

### 2.6 ELISA

The cultured medium of mouse primary keratinocytes was collected after stimulated with *Der f1* (10 μg/mL) or SLIGRL (100 μM) over a time course. MIF in the medium was measured using a mouse MIF ELISA kit (Elabscience) following the manufacturer’s instruction. This ELISA kit has the sensitivity of 9.38pg/mL and the detection range of 15.63-1000 pg/mL. The cultured media were diluted to 1:10 before the measurement.

### 2.7 Co-immunoprecipitation

Co-immunoprecipitation was conducted using a magnetic IP/co-IP kit (Invitrogen) according to the standard protocol. Protein A-G-magnetic beads were incubated with anti-KIF13B antibody (3 μg, Invitrogen) or anti-IgG antibody (3 μg, Invitrogen) at 4°C overnight. After adding cell lysates (1 mg of protein), each reaction was incubated at room temperature for 2 hours. The beads were washed with IP elution buffer. Cell lysates were separated on a 4-12% Bis-Tris NuPage gel (Invitrogen) and transferred onto a nitrocellulose filter membrane. The interaction was visualized using anti-P115 antibody (1:1000, Proteintech).

### 2.8 Multiplex immunohistochemistry

Co-staining of CD86 (1:200, Cell Signaling Technology) and CD163 (1:300, Abcam) was performed using a three-color mIHC fluorescence kit (Hunan Aifang Biotechnology) based on tyramide signal amplification technology according to the manufacturer’s introduction.

### 2.9 Statistical analyses

Statistical comparisons were performed using GraphPad Prism (version 10.0) with Student’s *t* test or one-way ANOVA as indicated (*P < 0.05; **P < 0.01; ***P < 0.001; ****P < 0.0001). Results are presented as mean ± SEM.

## 3 Results

### 3.1 The HDM-allergic AD mouse model shows a significant elevation of MIF in keratinocytes

**Figure 1:**
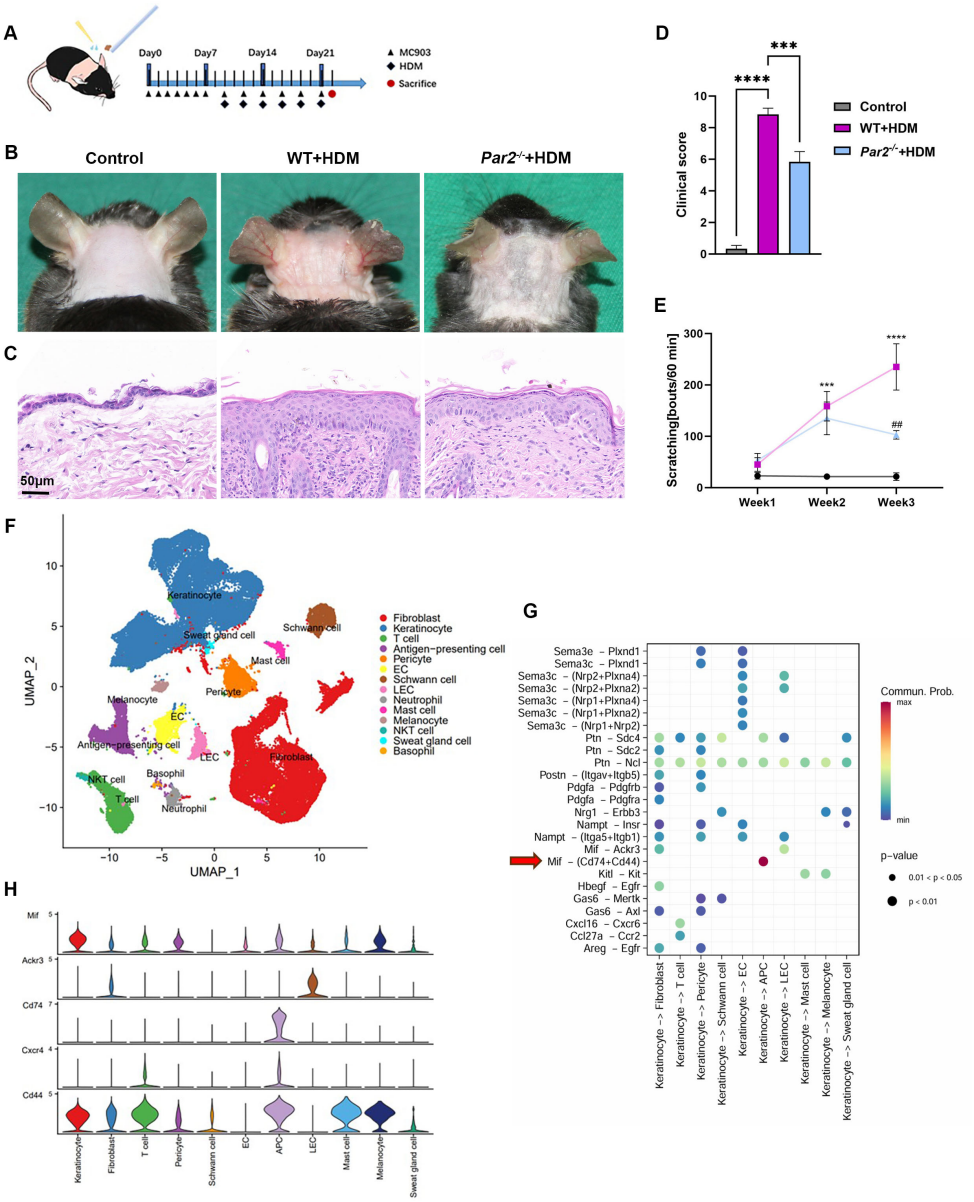
MIF expression is increased in HDM-allergic AD mouse model and is regulated by PAR2. **(A)** Mouse neck skin (2.5cm x 2.5cm) was topically applied with MC903 once a day for one week, and HDM ointment was then applied following MC903 every other day for 6 times. **(B)** Skin appearance showed that HDM-WT mice displayed serious erythema, edema and excoriation, and milder skin changes were found in HDM-*Par2^-/-^* mice. **(C)** H&E staining of skin sections showed histopathology changes of corresponding mice in panel **(B)**. Bar=50μm. **(D)** Clinical scores (redness, bleeding and eruption, each scaled 0 to 3) of the mice. One-way ANOVA, n=6/group, ***P<0.001, ****p < 0.0001. **(E)** Scratching bouts of mice in 60 minutes at week 1, week 2 and week 3 were more in HDM-WT mice than in HDM-*Par2^-/-^* mice. One-way ANOVA followed by Tukey’s *post hoc* test, n=6/group, ***p < 0.001, **** p < 0.0001, vs. control group; #p < 0.01 vs. HDM-WT group. **(F)** Uniform manifold approximation and projection (UMAP) plot of the single cell RNA sequencing results of the neck skins from WT control (n=2), HDM-WT (n=2) and HDM*-Par2^-/-^* (n=2), revealing 14 cell populations. **(G)** Predicted interaction of ligand-receptor between keratinocytes and dermal cells in HDM-WT mice. **(H)** MIF related signal molecules expressed in different cell types.

HDM derived allergens are important causative factors in the development of AD (1). To establish a HDM-allergic AD model in C57BL/6J mice, we first applied MC903 on mice shaved neck to induce a skin barrier dysfunction and to make it easier for external allergens entering into skin, then HDM ointment containing *Dermatophagoides farinae1* (*Der f1*) was topically applied to the same area (**Figure 1A**). The HDM group showed severe redness, swelling and eruption on the skin area compared to the control group (**Figures 1B, D**). Skin pathology showed significant hyperkeratosis and inflammatory cells infiltration in dermis of HDM group (**Figure 1C**). In addition, mice scratching bouts significantly increased in HDM-allergic AD mice (**Figure 1E**). We also set a control group applied with MC903 only, which showed slight pathological changes and pruritus sensation compared to MC903+HDM mice (**Supplementary Figures S1A–C**). Therefore, we have established a HDM-allergic AD mouse model with typical AD characteristics.

ScRNA-seq analysis was conducted for the skins from control mice (n=2), HDM-WT mice (n=2) and HDM-*Par2^-/-^* mice (n=2), and obtained a total of 68,939 single-cell transcriptome data of high-quality. UMAP for dimension reduction to visualize clustering was used, and a total of 14 cell types could be defined in skin samples: fibroblast, keratinocyte, T cell, antigen-presenting cell (APC), pericyte, endothelial cells (EC), Schwann cell, lymphatic endothelial cell (LEC), neutrophil, mast cell, melanocyte, NKT cell, sweat gland cell and basophil (**Figure 1F**). Then we employed CellChat function of Seurat to investigate the complicate cellular communication network; the results showed that the communication between the ligand MIF from keratinocytes and the receptors of Cd74 and Cd44 on APCs was the strongest (**Figures 1G, H**). In other words, keratinocytes secrete MIF after they contact with the external antigens of HDM, and the MIF as a ligand binds to the receptors of Cd74 and Cd44 on the APCs nearby the keratinocytes to initiate the immunologic responses of AD.

To further exclude the role of the MC903 in HDM-allergic AD model, we also employed oxazolone, a hapten to induce contact dermatitis, combined with HDM to establish a HDM-allergic AD model (**Supplementary Figure S2A**). Oxazolone+HDM mice displayed severer AD-like symptoms, hyperkeratosis and lymphocyte infiltration, and higher MIF mRNA and stronger MIF immunofluorescence signals in skin, similar to MC903+HDM mice; while oxazolone without HDM mice showed slight AD-like presentations (**Supplementary Figures S2B–G**), indicating that *Der f1* rather than MC903 is the main allergen to produce the AD model.

### 3.2 PAR2 regulates MIF expression in keratinocytes and MIF secretion from keratinocytes of the HDM-allergic AD model

**Figure 2:**
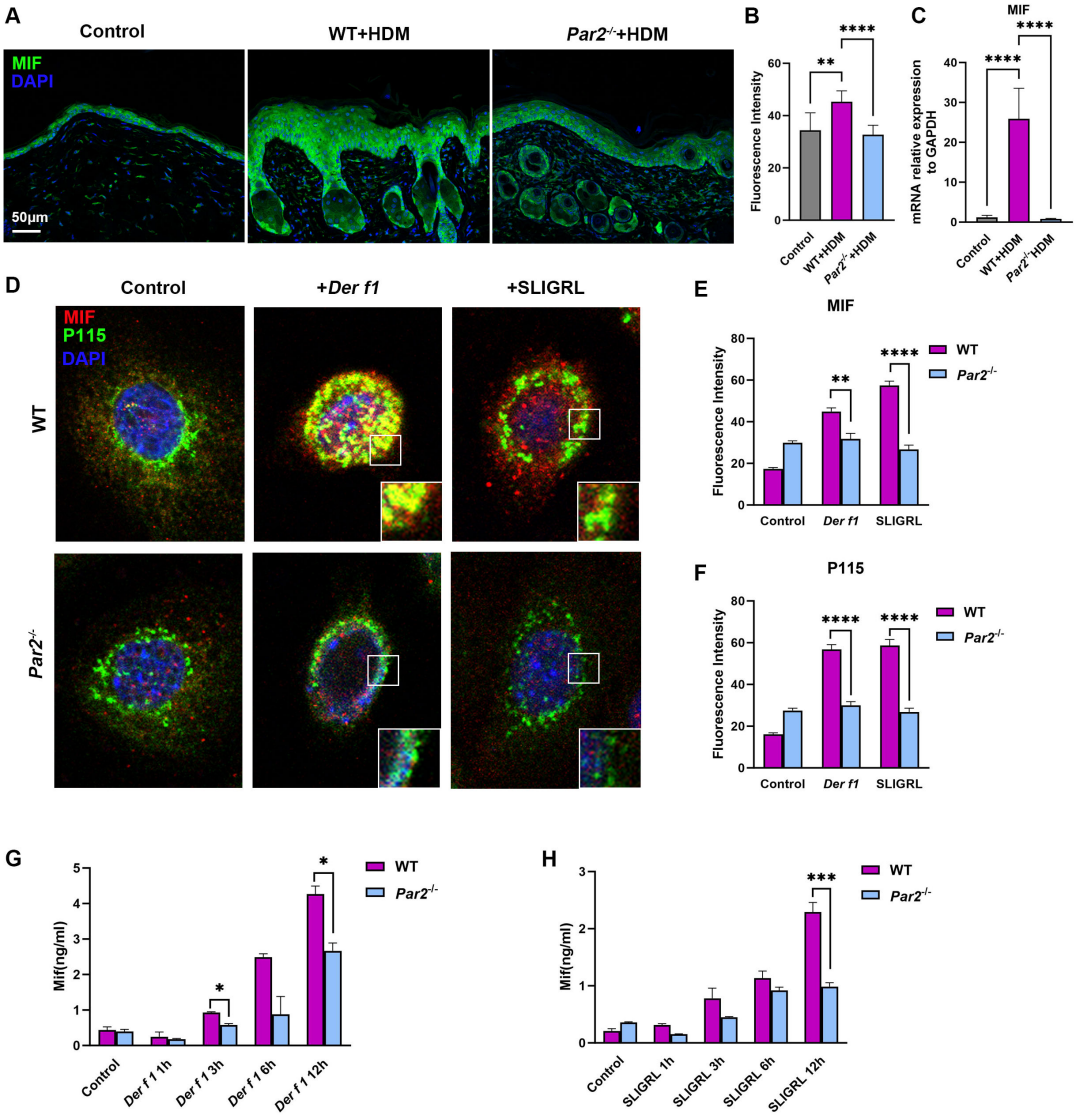
PAR2 activation increases MIF expression in keratinocytes and secretion from keratinocytes. **(A, B)** Immunofluorescence staining of neck skin sections and quantitative analysis of the fluorescence signals showing higher MIF expression in HDM-WT skin than in HDM*-Par2^-/-^* skin. Bar=50μm. One-way ANOVA, **p< 0.01, ****p < 0.0001. **(C)** RT-PCR of MIF mRNA relative to GAPDH mRNA in neck skins. One-way ANOVA, n=3-4/group, ****p < 0.0001. **(D–F)** Cellular immunofluorescence and quantitative analysis of the fluorescence signals showed that the expressions of MIF and P115 were higher in WT keratinocytes than in *Par2^-/-^* keratinocytes after stimulation with *Der f1* or SLIGRL, and that MIF and P115 were co-localized in the cells. Unpaired *t* tests, **p< 0.01, ****p < 0.0001. **(G)** ELISA of MIF in the cultured media of WT keratinocytes and *Par2^-/-^* keratinocytes after stimulation with *Der f1* for 12h. More MIF released from WT keratinocytes than from *Par2^-/-^* keratinocytes. Unpaired *t* tests, *P < 0.05. **(H)** ELISA of MIF in the cultured media of WT keratinocytes and *Par2^-/-^* keratinocytes after stimulation with SLIGRL for 12h. More MIF released from WT keratinocytes than from *Par2^-/-^* keratinocytes. Unpaired *t* tests, ***P < 0.001.

The protease activity of HDM may activate PAR2 on keratinocytes. We therefore investigated the effects of PAR2 on the HDM-allergic AD model. The HDM-allergic AD model was also established in *Par2^-/-^* mice, and found that HDM-*Par2^-/-^* mice displayed milder erythema, swelling and eruption, less hyperkeratosis and lymphocyte infiltration, and a decrease number of scratch bouts compared to HDM-WT mice (**Figures 1B–E**). Immunofluorescence and RT-PCR confirmed that MIF and its mRNA in skin were higher in HDM-WT mice and much lower in HDM*-Par2^-/-^* mice (**Figures 2A–C**). Primary keratinocytes from WT and *Par2^-/-^* mice were cultured and stimulated with *Der f1*, and SLIGRL, a PAR2 agonist. *Der f1* and SLIGRL stimulation caused higher expression of MIF in mouse keratinocytes by immunofluorescence (**Figures 2D–F**). Moreover, *Der f1* and SLIGRL stimulation induced more MIF released from WT keratinocytes than from *Par2^-/-^* keratinocytes by ELISA (**Figures 2G, H**).

### 3.3 PAR2 is involved in the intracellular trafficking and secretion of MIF through regulating the expressions of KIF13B and P115 and acetylation of α-tubulin in keratinocytes

MIF has no N-terminal signal peptide for trafficking in endoplasmic reticulum (14). MIF is therefore secreted in a non-classical mechanism that does not depend on signal peptide cleavage (14). Golgi vesicle transporter protein P115 can serve as a cofactor to help MIF secretion (15). Immunofluorescence of primary keratinocytes and skin sections found that MIF and P115 expression increased and co-localized in the samples from HDM-WT mice, and decreased in the samples from HDM-*Par2^-/-^* mice (**Figure 2D**, **Supplementary Figure S3**).

**Figure 3:**
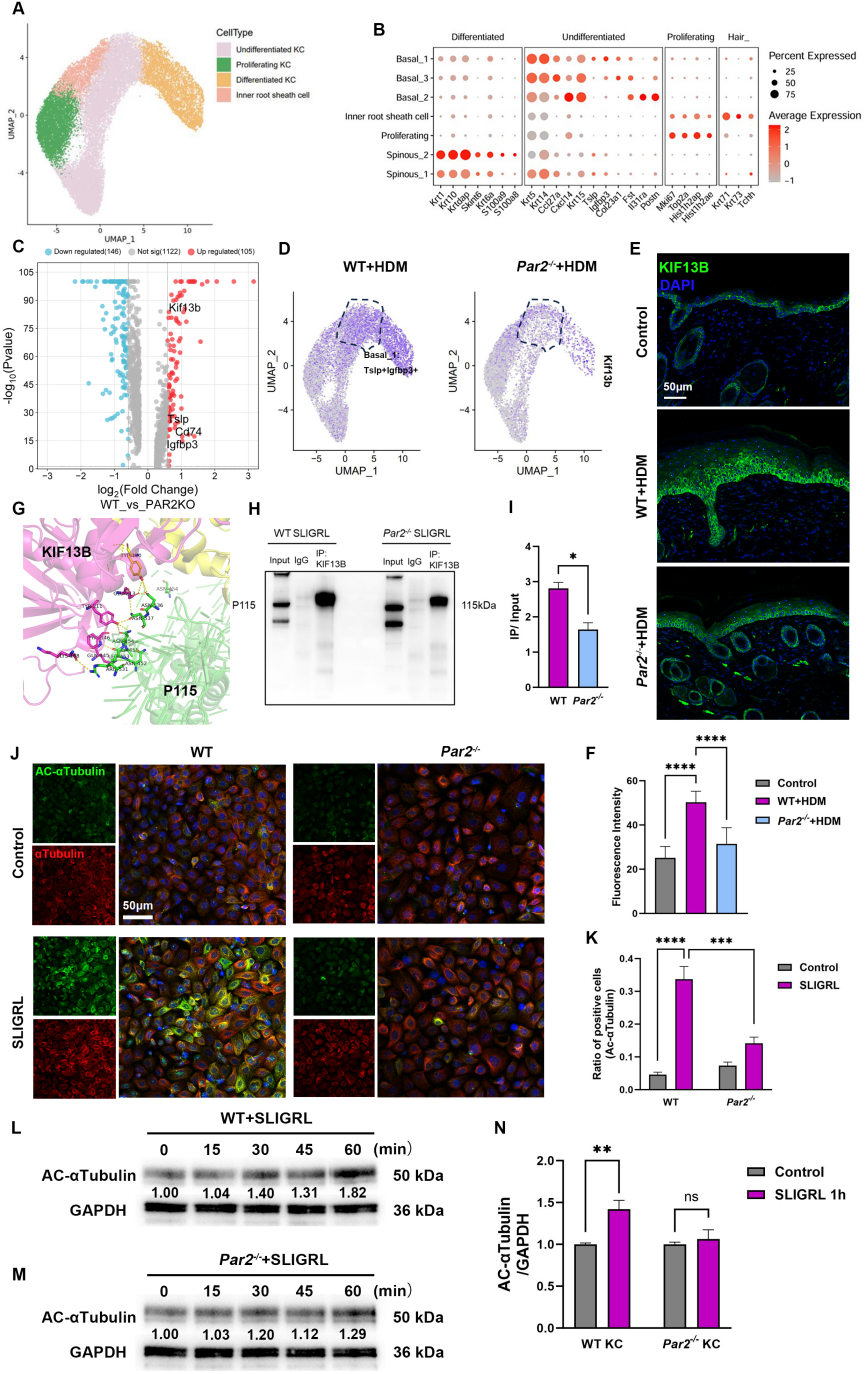
The regulation of MIF release by PAR2 is associated with the increases of KIF13B expression and α-tubulin acetylation. **(A)** UMAP plot of keratinocyte subsets revealing 4 cell populations. **(B)** Dot plot displays marker genes in each cell population. **(C)** Volcano plot of differentially expressed genes in basal_1 clusters from HDM-WT samples and from HDM-*Par2^-/-^* samples. **(D)** UMAP plot showed the expression of KIF13B increased more in TSLP^+^/IGFBP3^+^ basal keratinocytes in HDM-WT samples than in HDM*-Par2^-/-^* samples. **(E, F)** Immunofluorescence images and quantitative analysis of the fluorescence signals of neck skin sections showed that KIF13B expression was more in HDM-WT mice than in HDM-*Par2^-/-^* mice. Bar=50μm. **(G)** Prediction of the reciprocal interaction regions of KIF13B (shown in magenta) and P115 (shown in green). **(H, I)** Co-immunoprecipitation and quantitative analysis of the positive signals showed that the integration of KIF13B and P115 in keratinocytes stimulated with SLIGRL was stronger in WT keratinocytes than in *Par2^-/-^* keratinocytes *P<0.05. **(J, K)** Cellular immunofluorescence showed that primary keratinocytes were stimulated with SLIGRL for one hour, and acetylated α-tubulin increased more in WT keratinocytes than in *Par2^-/-^* keratinocytes. Bar=50μm. Unpaired *t* tests, ***P < 0.001, ****P < 0.0001. **(L–N)** Primary keratinocytes were stimulated with SLIGRL for 15, 30, 45 and 60 min, and western blot showed that acetylated α-tubulin was higher in WT keratinocytes than in *Par2^-/-^* keratinocytes. Unpaired *t* tests, **P < 0.01, ns, no significance.

To determine the specific subsets of keratinocytes for PAR2-related MIF release process, the scRNA-seq data from control, HDM-WT and HDM-*Par2^-/-^* groups were further analyzed. Keratinocytes can be divided into 4 distinct clusters: undifferentiated keratinocyte (KC), differentiated KC, proliferating KC and inner root sheath cell (**Figure 3A**, **Supplementary Table S2**). Basal_1, a subtype of undifferentiated KC, was characterized by the expression of TSLP and IGFPB3, and was important in inflammation in AD development (**Figure 3B**). We also calculated differentially expressed genes (DEGs) from scRNA-seq data to compare basal_1 subtype in HDM-WT group against that in HDM-*Par2^-/-^* group and revealed the higher expression of Cd74, TSLP and IGFBP3 in HDM-WT group, indicating the participation of basal keratinocytes in the inflammation and immunomodulation of AD (**Figure 3C**).

Notably, DEGs also showed that KIF13B, a kinesin protein, was highly expressed in basal_1 subtype of HDM-WT group (**Figures 3C, D**). Using immunofluorescence assay, we confirmed the higher expression of KIF13B in keratinocytes in HDM-WT group than those in HDM*-Par2^-/-^* group (**Figures 3E, F**). We also proved that KIF13B, P115 and MIF were co-localized and highly expressed in HDM-allergic WT model by mIHC assay (**Supplementary Figure S4**). Protein docking assay predicted the existence of reciprocal interaction regions in KIF13B and P115 (**Figure 3G**). P115 and KIF13B were co-immunoprecipitated in SLIGRL treated primary keratinocytes of WT mouse, but the co-immunoprecipitation was weaker in those of *Par2^-/-^* mouse (**Figures 3H, I**).

KIF13B has a motor domain anchored on the microtubules and a tail region binding to the cargo to mediate transport of cargos on microtubules (16). We also found that acetylation of α-tubulin increased after stimulation by SLIGRL in WT keratinocytes but not in *Par2^-/-^* keratinocytes by immunofluorescence (**Figures 3J, K**) and western blotting (**Figures 3L–N**). In addition, acetylated α-tubulin protein increased in a time-dependent manner after SLIGRL stimulation in WT but not in *Par2^-/-^* keratinocytes (**Figures 3L–N**). These findings indicate PAR2 regulates the expression of KIF13B and the acetylated α-tubulin in keratinocytes to affect the intracellular trafficking of MIF.

### 3.4 *In vivo* and *in vitro* experiments using MIF antagonist ISO-1 demonstrates that MIF is an important cytokine in the pathogenesis of AD-like changes in HDM-allergic AD model

**Figure 4:**
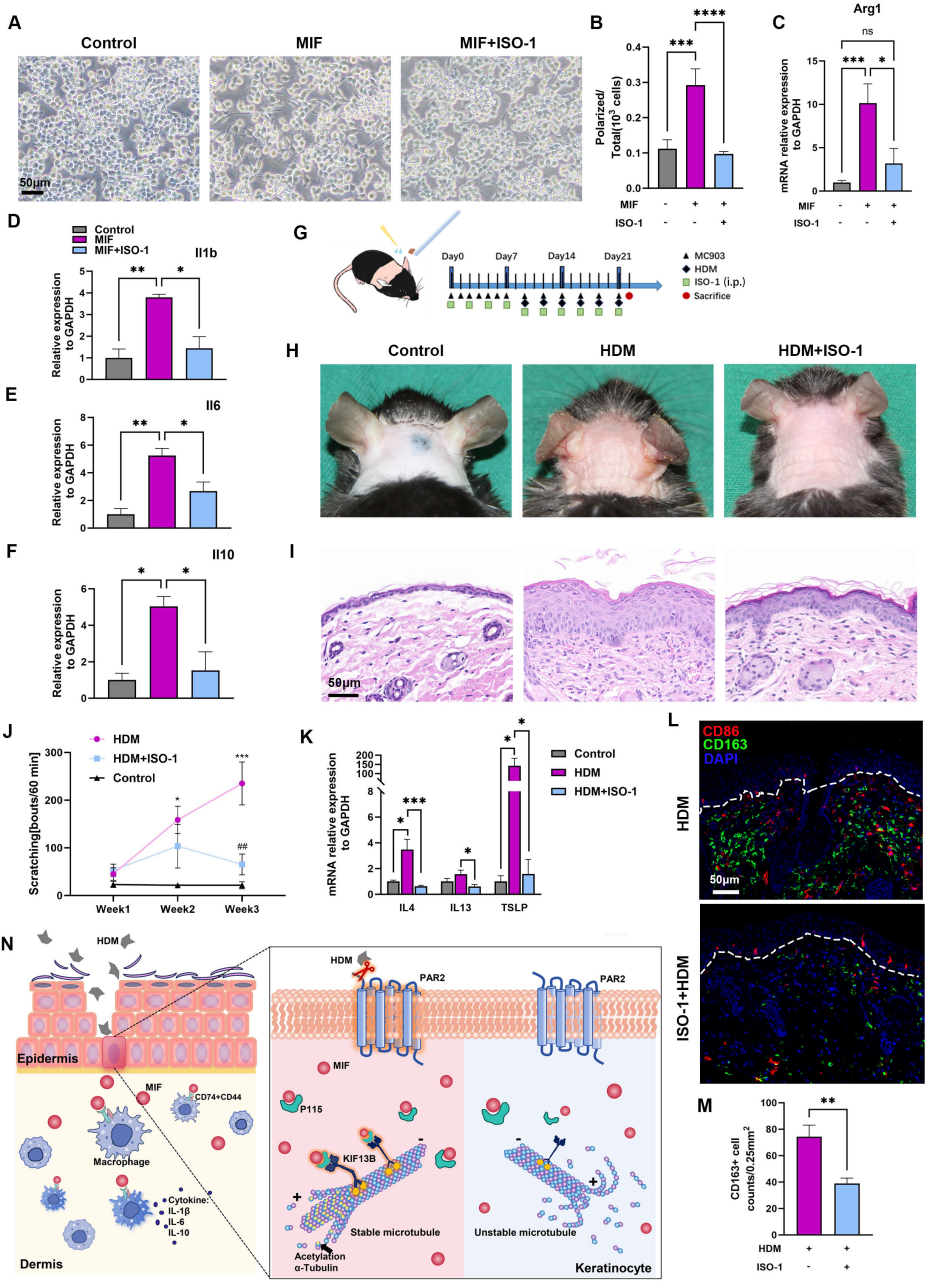
MIF antagonist ISO-1 relieves AD presentations and macrophage activation in HDM-allergic AD model. **(A, B)** Polarized change and its quantitative analysis of Raw264.7 cells stimulated with MIF or MIF+ISO-1. One-way ANOVA, ***p<0.001, **** p<0.0001. Bar=50μm. **(C)** RT-PCR showed that Arg1 mRNA expression increased in Raw264.7 cells stimulated with MIF, and Arg1 mRNA expression was inhibited in Raw264.7 cells treated with MIF+ISO-1 One-way ANOVA, *p<0.05, *** p<0.001. "ns, no significance. **(D–F)** RT-PCR showed that IL-1b, IL-6 and IL-10 mRNA levels relative to GAPDH mRNA increased in Raw264.7 cells after MIF treatment for 24 hours, and decreased in the cells after MIF+ISO-1 treatment for 24 hours. One-way ANOVA, *p<0.05, ** p<0.01. **(G)** Mouse neck skins were topically applied with MC903 once a day at first week, and HDM ointment was then applied following MC903 every other day for 6 times. ISO-1 (35mg/kg) was injected intraperitoneally every other day for 6 times. **(H)** Skin lesions showed that ISO-1 relieved erythema, edema, and excoriation in the AD model. **(I)** Histopathology changes of corresponding mice in panel **(H)** Bar=50μm. **(J)** Scratching behaviors in 60 minutes decreased after ISO-1 treatment at week 1, week 2 and week 3. One-way ANOVA followed by the Tukey’s *post hoc* test, n=4-6/group, *p < 0.05, *** p < 0.001 vs. control group; ##p < 0.01 vs. HDM group. **(K)** RT-PCR showed that IL-4, IL-13 and TSLP mRNA levels relative to GAPDH mRNA decreased in the skins of HDM-WT+ISO-1 mice as compared to those in the HDM-WT mice. Unpaired *t* tests, n = 3-5, *P<0.05, ***P<0.001. **(L, M)** Representative images and quantitative analysis of multiplex immunohistochemistry in neck skins showed that there were less CD163+ macrophages in HDM-WT+ISO-1 mice. Unpaired *t* tests, **P < 0.01. Bar=50μm. **(N)** Diagram of PAR2-MIF axis in skin participating in macrophage polarization and activation in HDM-allergic AD model.

The key role of MIF in the pathogenesis of AD was verified by the stronger communication between ligand MIF from keratinocytes and receptors Cd74 and Cd44 on APCs from the results of scRNA-seq, the higher immunofluorescence signals of MIF in HDM-WT skin, and the increased MIF secretion from keratinocytes after *Der f1* stimulation. MIF antagonist ISO-1 was then used to observe its effects on HDM-allergic AD model. We first tested the usefulness of ISO-1 by stimulation of MIF or MIF+ISO-1 to cultured mouse macrophage cell line Raw264.7 cells. Cellular polarization such as horns and adherence and the expression of Il-1B, IL-6 and IL-10 mRNAs by RT-PCR were increased in Raw264.7 cells stimulated by MIF, and these changes were significantly inhibited by MIF+ISO-1 (**Figures 4A–F**). Moreover, Arg1 mRNA, a biomarker of M2 macrophage, decreased markedly after ISO-1 treatment by RT-PCR (17) (**Figure 4C**). Intraperitoneal injection of ISO-1 (35mg/kg) was then used every other day to HDM-allergic AD mice (**Figure 4G**). After ISO-1 treatment, the mice displayed milder redness, swelling and eruption, improved hyperkeratosis in epidermis and inflammatory cells infiltration in dermis, decreased number of scratching bouts (**Figures 4H–J**), associated with the decreases of the type 2 related cytokine mRNAs, including IL-4, IL-13 and TSLP mRNAs by RT-PCR (**Figure 4K**), and the decrease of CD163^+^ M2 macrophage infiltration by immunofluorescence (**Figures 4L, M**) in the neck skin of HDM-WT mice. Therefore, treatment of ISO-1 to HDM-WT mice achieved significant improvement of AD symptoms.

## 4 Discussion

We used interdisciplinary approaches to disclose the PAR2-MIF axis in the pathogenesis of AD in HDM-allergic AD model. HDM activates PAR2, which promotes MIF secretion from the keratinocytes, and the secreted MIF triggers a series of immunologic and inflammatory responses of AD. To the best of our knowledge, this study provides the first evidence that the MIF antagonist exhibits a satisfactory therapeutic effect on AD-like presentations in HDM-allergic AD model.

Epidermal PAR2 overexpressed transgenic mice treated with HDM exhibits typical characterizations of AD (4, 18). We demonstrated that *Par2^-/-^* mice displayed milder symptoms and skin pathology than WT mice in HDM-allergic AD model. The dust mite allergens have immunogenicity as well as cysteine and serine protease activities. *Der f1* protease, the main component in HDM ointment, up-regulates the expressions of IL-6, IL-8 and IL-33 mRNAs and GM-CSF release from keratinocytes via PAR2 activation (6, 19). HDM proteases can also activate PAR2 in airway epithelial cells to generate activation signals and mobilization of intracellular Ca^2+^ (7). We found that *Der p1* (another common allergen in environment) rather than *Der f1* could activate PAR2 to induce Ca^2+^ mobilization, and PAR2 antagonist FSLLRY peptide inhibited the Ca^2+^ mobilization in HaCaT cells (**Supplementary Figure S5**). Toll-like receptors (TLRs) are important pattern-recognition receptors (PRRs) in innate immune system. For example in inflammation, neutrophil elastase cleaves and activates PAR2, which then triggers distinct signaling cascades to trans-activate TLR4 in macrophage (20). The PAR-TLR interactions also exist in epithelial cells (21). Blocking TLR4 with its antagonist TAK-242 inhibited *Der f1*-induced MIF release in mouse primary keratinocytes (**Supplementary Figure S6**), suggesting the importance of TLR4 in the cascade of PAR2-TLR4-MIF secretion in HDM stimulated keratinocytes.

PAR2 activation induced MIF secretion is associated with the increase of P115 expression in a non-inflammatory mechanism of insulin resistance (12). We found that PAR2 activation induced the increases of P115 expression in keratinocytes and MIF release from keratinocytes. MIF is necessary for neuropathic and inflammatory pain following nerve injury (22). However, whether MIF is involved in itching sensation is yet unknown. Our results showed that MIF combined with the PAR2 agonist SLIGRL produced acute pruritus flares (**Supplementary Figure S7**), indicating that MIF may also directly mediate itch sensation.

Analysis of our scRNA-seq results identified a TSLP^+^/IGFBP3^+^ basal keratinocyte subset higher expressed in HDM-WT mice than in HDM-*Par2^-/-^* mice. PAR2 activation has been reported to induce TSLP expression in keratinocytes, and TSLP is a “switch” initiating Th2-type allergic inflammatory responses (23). IGFBP3 is a key protein in insulin like growth factor (IGF) pathway and is highly expressed in AD children (24). AD in children is often considered to be the first step of atopic march. Therefore, TSLP^+^/IGFBP3^+^ basal keratinocytes may be the keratinocyte subset taking part in the inflammatory signaling and disease exacerbation in AD. KIF13B functions in axon transportation to mediate axon development in neurons (25), and is highly expressed in TSLP^+^/IGFBP3^+^ keratinocytes in HDM-WT mice. The Thr-506 phosphorylation of KIF13B activates the transport of TRPV1-containing vesicles to membrane, contributing to heat sensitivity (26). Our data demonstrate that the binding of KIF13B and P115 to MIF mediates MIF secretion from keratinocytes to produce immunologic and inflammatory reactions in HDM-allergic AD model.

Lys-40 acetylation of α-tubulin is the sole posttranslational modification of microtubules to affect microtubule stability and function (27), and is positively regulated by ATAT1 and negatively regulated by HDAC6 (28). PAR2 activation regulated by TRPA1 promotes the transport of melanosome in keratinocytes via modulating acetylation of α-tubulin (29), consistent with our results that PAR2 activation promoted acetylation of α-tubulin and thereby maintained microtubule stability and intracellular trafficking of MIF in keratinocytes.

M2 macrophages are essential to allergen clearance and to provide a Th2-dominated immune milieu in AD by secretion of chemokines and cytokines (30, 31). MIF regulates the polarization towards M2 phenotype and induces a dynamic shift from M1 to M2 phenotype in THP-1 macrophages (32, 33). MIF also recruits peritoneal inflammatory macrophages for pathogens phagocytosis in an experimental poly-microbial sepsis model (34). In our HDM-allergic AD model, substantial infiltration of M2 macrophages in dermis was found, and the infiltration was inhibited by MIF antagonist ISO-1. The infiltrated M2 macrophages may function as APCs, presenting antigens to activate T cells and amplify allergic responses. Therefore, the PAR2-MIF axis in skin is important in polarization of M2 macrophages and initiation of immunologic and inflammatory responses in AD.

The efficacy of PAR2 antagonists in AD animal models is well verified (13), but the clinical application is yet limited. MEDI0618 is a PAR2 antibody and has been taken into phase I clinical trial (NCT04198558) in healthy volunteers. Our and other researches have revealed the satisfactory therapeutic effects of antagonizing MIF in AD mouse models and other allergy-related diseases (35). Several phase I clinical trials of anti-MIF monoclonal antibody imalumab and anti-CD74 monoclonal antibody milatuzumab showed well-tolerance and efficacy in cancer (NCT01765790, NCT02540356 and NCT02448810), systemic lupus erythematosus (NCT01845740) and hematologic disorders (NCT01101594, NCT00603668 and NCT00989586). Therefore, targeting PAR2 and MIF is promising in the treatment of HDM-allergic AD.

In conclusion, the release of MIF mediated by PAR2 activation is a crucial process relating to the immune imbalance and pruritus found in HDM-allergic AD model. PAR2 regulates MIF secretion by modulating the binding to KIF13B and acetylation of α-tubulin; the secreted MIF induces polarization and activation of macrophages (**Figure 4N**). Our findings underscore the significance of the PAR2-MIF axis, offering novel therapeutic targets for management of AD.

## Data Availability

The scRNA-seq data presented in this study are deposited in the GEO repository, accession number is http://www.ncbi.nlm.nih.gov/bioproject/1149532. All data generated or analyzed during this study are included in this article and its online **Supplementary Material.**
