# Supplementary material for: The role of PAR2 in regulating MIF release in house dust mite-induced atopic dermatitis
Source: Front Immunol. 2024 Oct 2;15:1478292. doi: 10.3389/fimmu.2024.1478292 (PMC11479884; doi:10.3389/fimmu.2024.1478292)
Supplement: Supplementary file 1 [file DataSheet1.docx]

**Supplemental Materials**


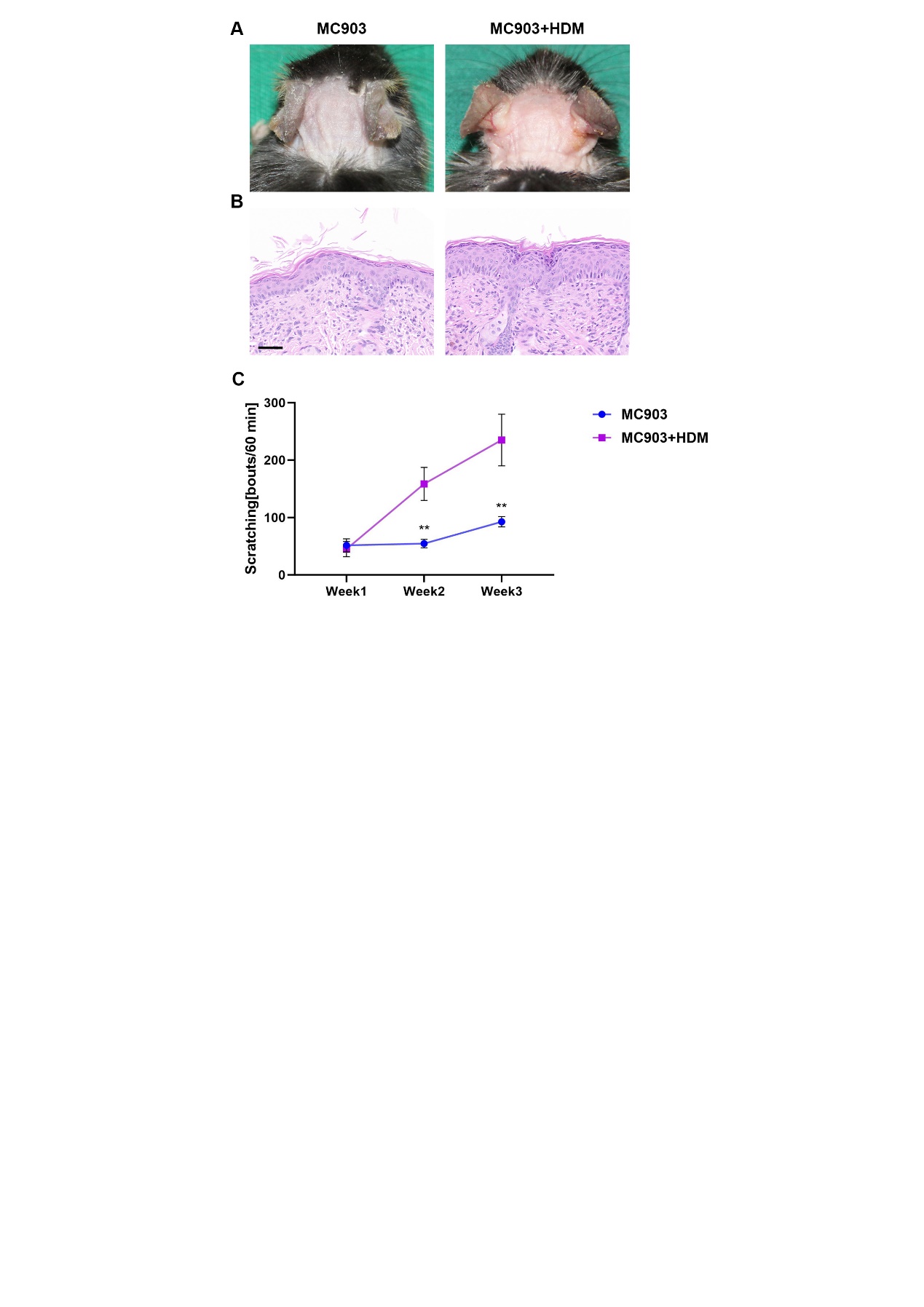


**Figure S1. MC903+HDM treated mice** **exhibit severer AD symptoms compared to MC903 treated mice.** **(A)** Skin appearance showed that MC903+HDM mice displayed more serious erythema, edema, and excoriation than MC903 mice. **(B)** H&E staining of skin sections showed that severer hyperkeratosis and inflammatory cell infiltration were found in MC903+HDM mice. Bar=50μm. **(C)** MC903+HDM mice had more scratching bouts in 60 minutes than MC903 mice. Unpaired *t* tests, **P < 0.01.


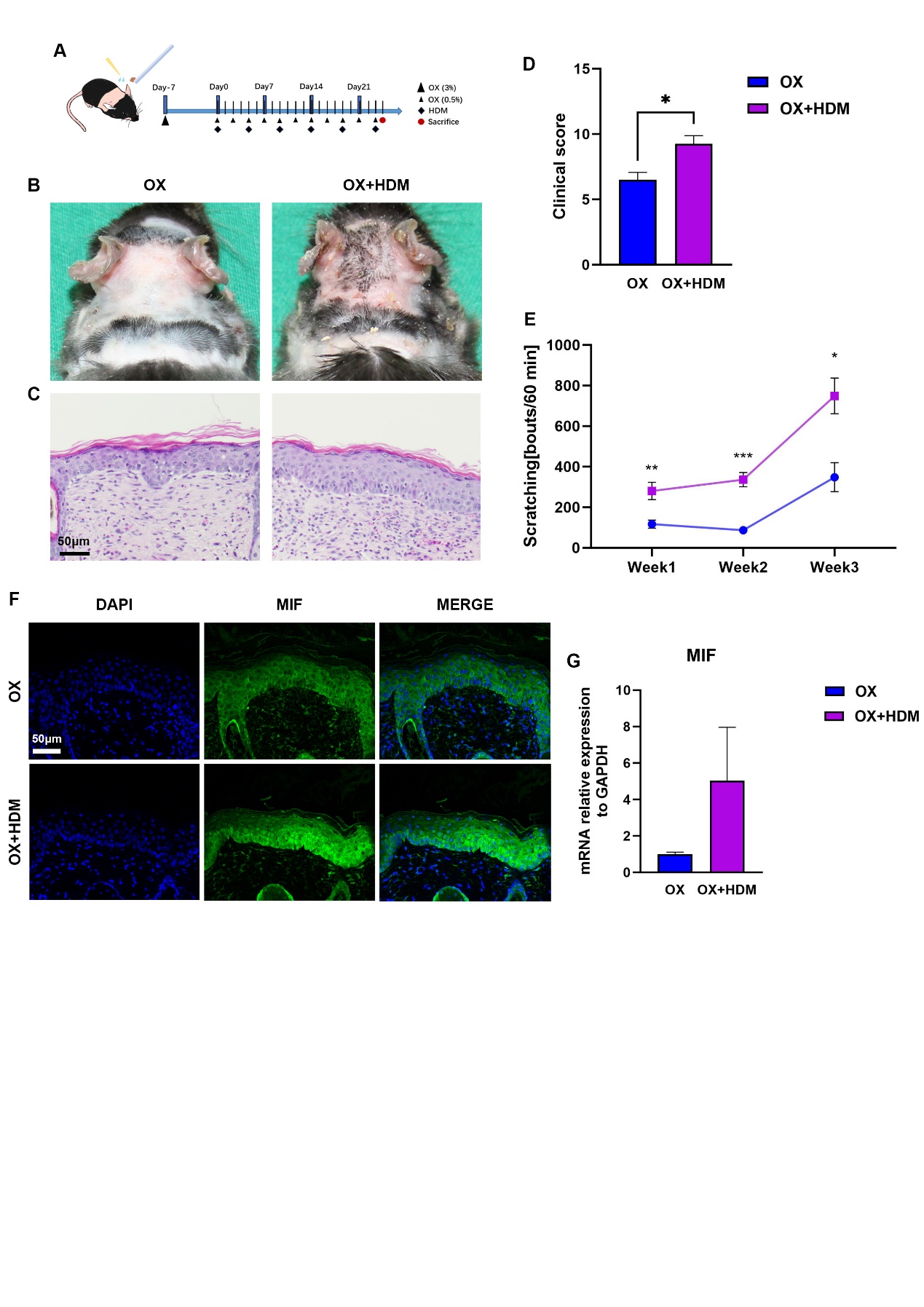


**Figure S2. MIF expression is upregulated in the OX+HDM treated AD model compared to OX treated mice.** **((A)** Mice neck skin (2.5cm*2.5cm) was topically applied once with oxazolone (3% in 4 acetone: 1olive oil). After 7 days, 0.5% oxazolone was topically applied every other day for three weeks. HDM ointment was applied twice per week for 6 times. **(B)** Skin appearance showed that OX+HDM mice displayed more serious erythema, excoriation and desquamation than OX mice. **(C)** H&E staining of skin sections showed that severer hyperkeratosis and inflammatory cell infiltration were found in OX+HDM mice. Bar=50μm. **(D)** Higher clinical score (redness, bleeding, eruption and scaling scored 0-3 each) was found in OX+HDM mice than in OX mice. Unpaired *t* tests, *P < 0.05. **(E)** More scratching bouts in 60 minutes were found in OX+HDM mice than in OX mice. Unpaired *t* tests, *P < 0.05, **P < 0.01, ***P < 0.001. **(F)** Representative immunofluorescence images of MIF in skins showed that OX+HDM mice expressed more MIF than OX mice. Bar=50μm. **(G)** MIF mRNA expression in skin was higher in OX+HDM treated mice than in OX treated mice.

OX, oxazolone.


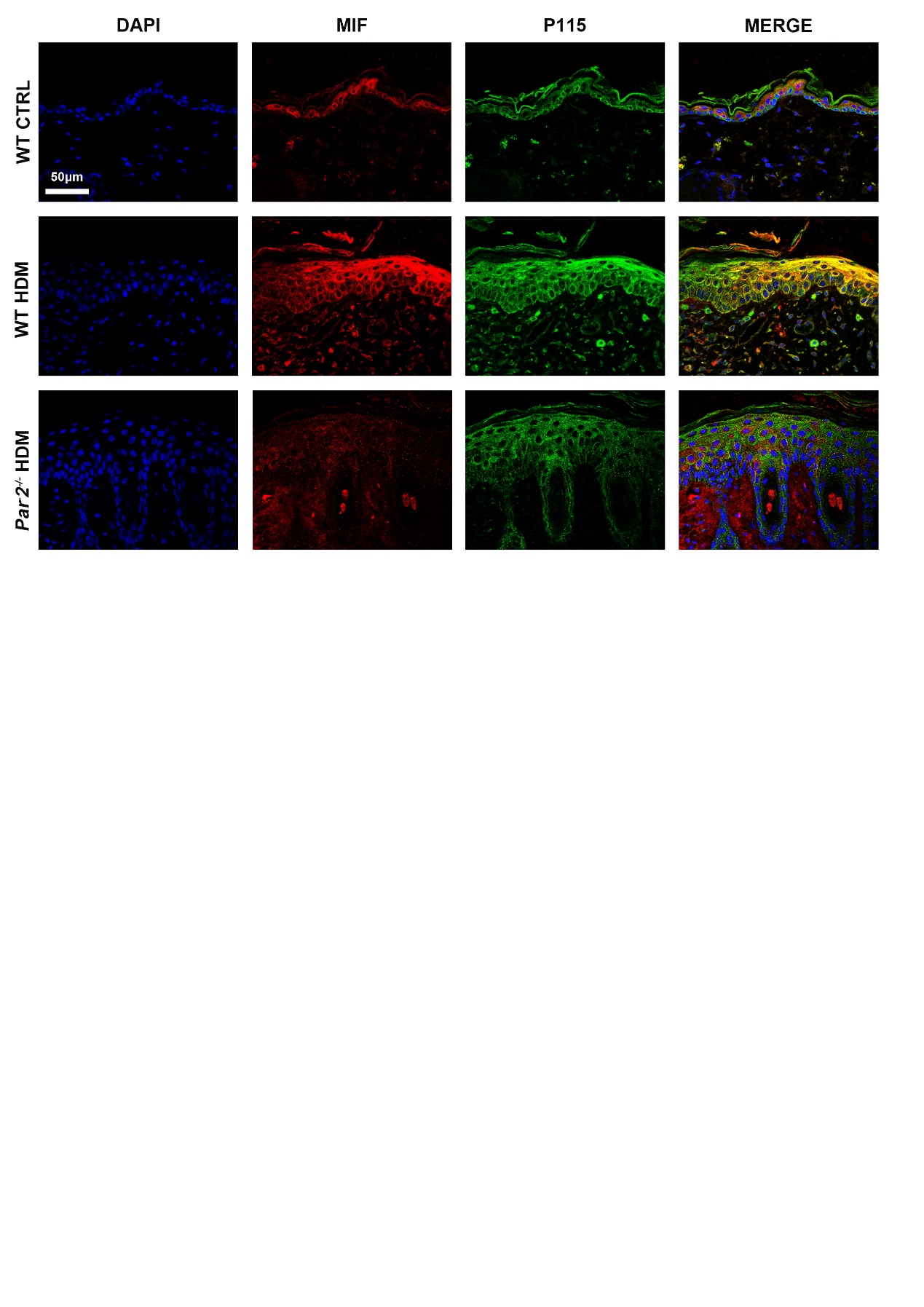


**Figure S3. Representative images of MIF and P115 expression in the skin of AD mice and control group.** Stronger MIF and P115 expressions and co-localization in skin were found in HDM-WT mice than in HDM-*Par2^-/-^* mice. Bar=50μm. WT, wild type. CTRL, control.


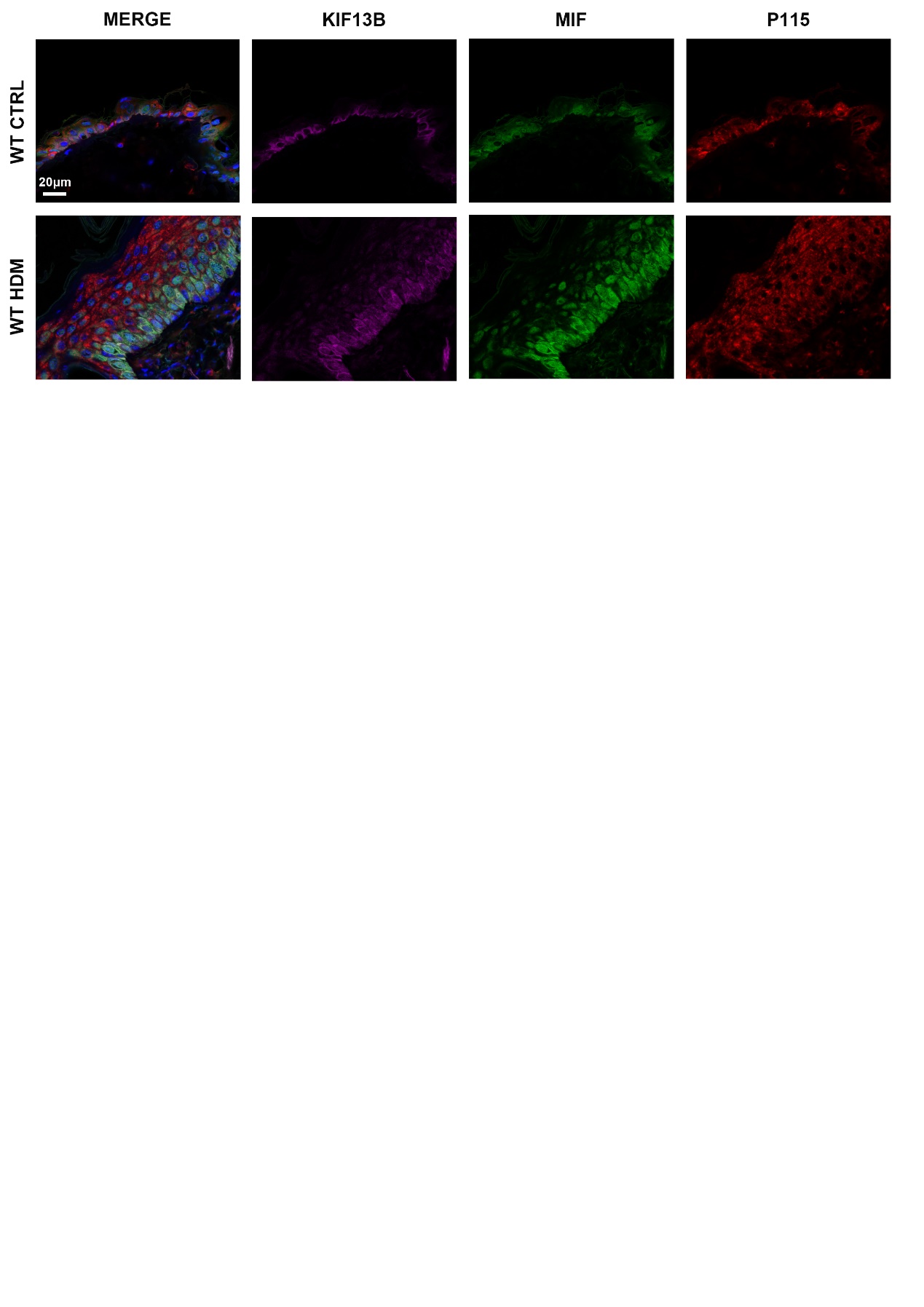


**Figure S4. Representative images of of KIF13B, MIF and P115 expression in the skin of AD mice and control group.** Stronger MIF expression and co-localization of KIF13B, MIF and P115 in skin were found in HDM-WT mice than in control group. . Bar=20μm.WT, wild type. CTRL, control.


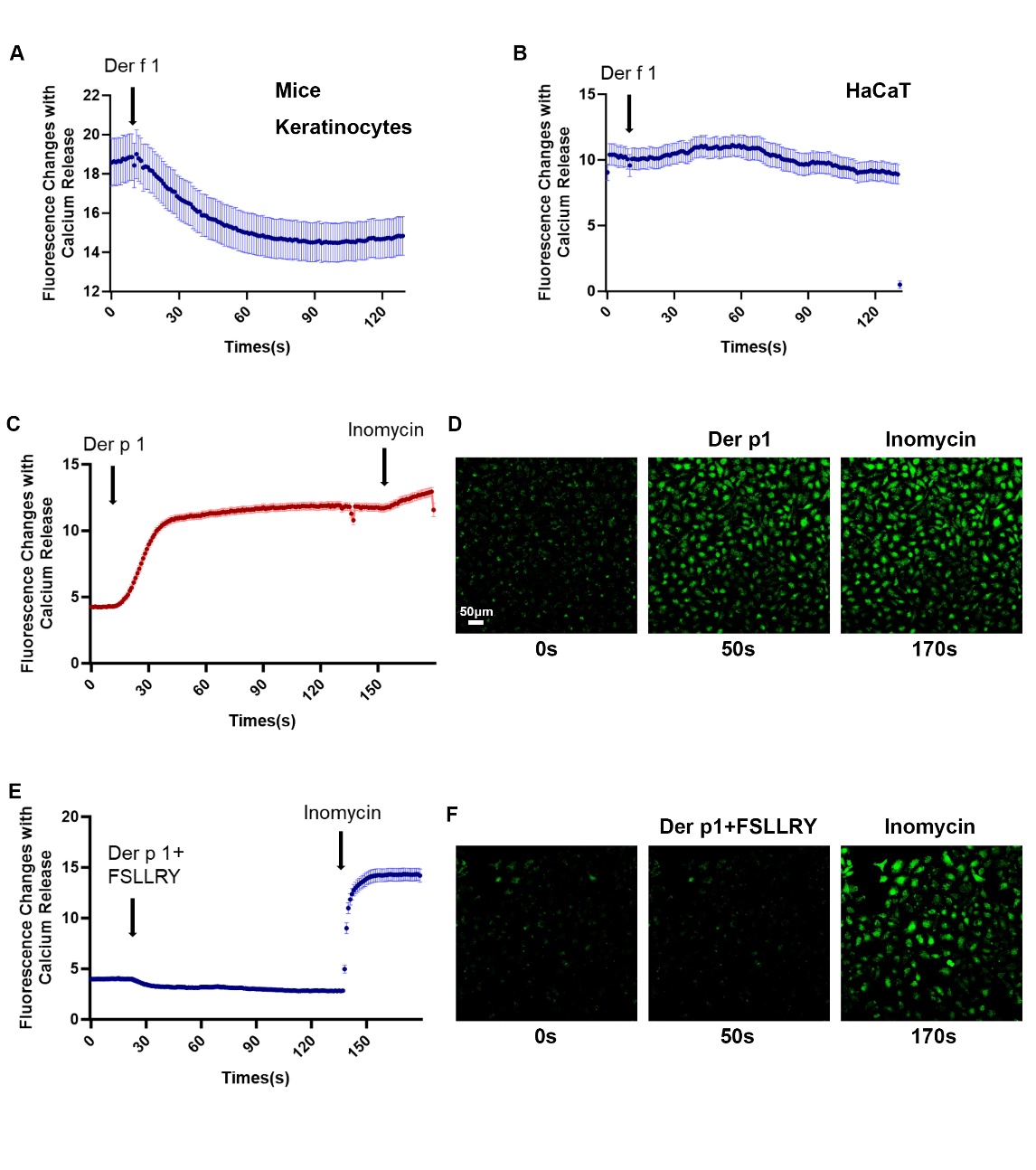


**Figure S5. Neither mice keratinocytes nor HaCaT cells exhibit Ca²⁺ mobilization in response to *Der f1*. In contrast, Der p1 successfully triggers Ca²⁺ mobilization in HaCaT cells, which can be inhibited by the PAR2 antagonist FSLLRY.** **(A)** Representative traces showed that there were no intracellular Ca^2+^ responses elicited by *Der f1* (20μg/μl) in mouse keratinocytes. **(B)** Representative traces showed that there were no intracellular Ca^2+^ responses elicited by *Der f1* (20μg/μl) in HaCaT cells. **(C)** Representative traces showed that Ca^2+^ mobilization was elicited by *Der p1* (25μg/μl) in HaCaT cells. Inomycin served as a positive control. **(D)** Representative fluorescence images of Fluo 4 (5 μM) loaded HaCaT cells showed the Ca^2+^ responses stimulated by *Der p1* at 0s, 50s and 170s. Bar=50μm. **(E)** Representative traces showed that the Ca^2+^ mobilization induced by *Der p1* (25μg/μl) in HaCaT cells was inhibited by pretreatment of FSLLRY (100μM). Inomycin served as a positive control. **(F)** Representative fluorescence images of Fluo 4 (5 μM) loaded HaCaT cells showed that the Ca^2+^ responses induced by *Der p1* was inhibited by *Der p1*+FSLLRY treatment at 0s, 50s and 170s. N = 3 mice per group.


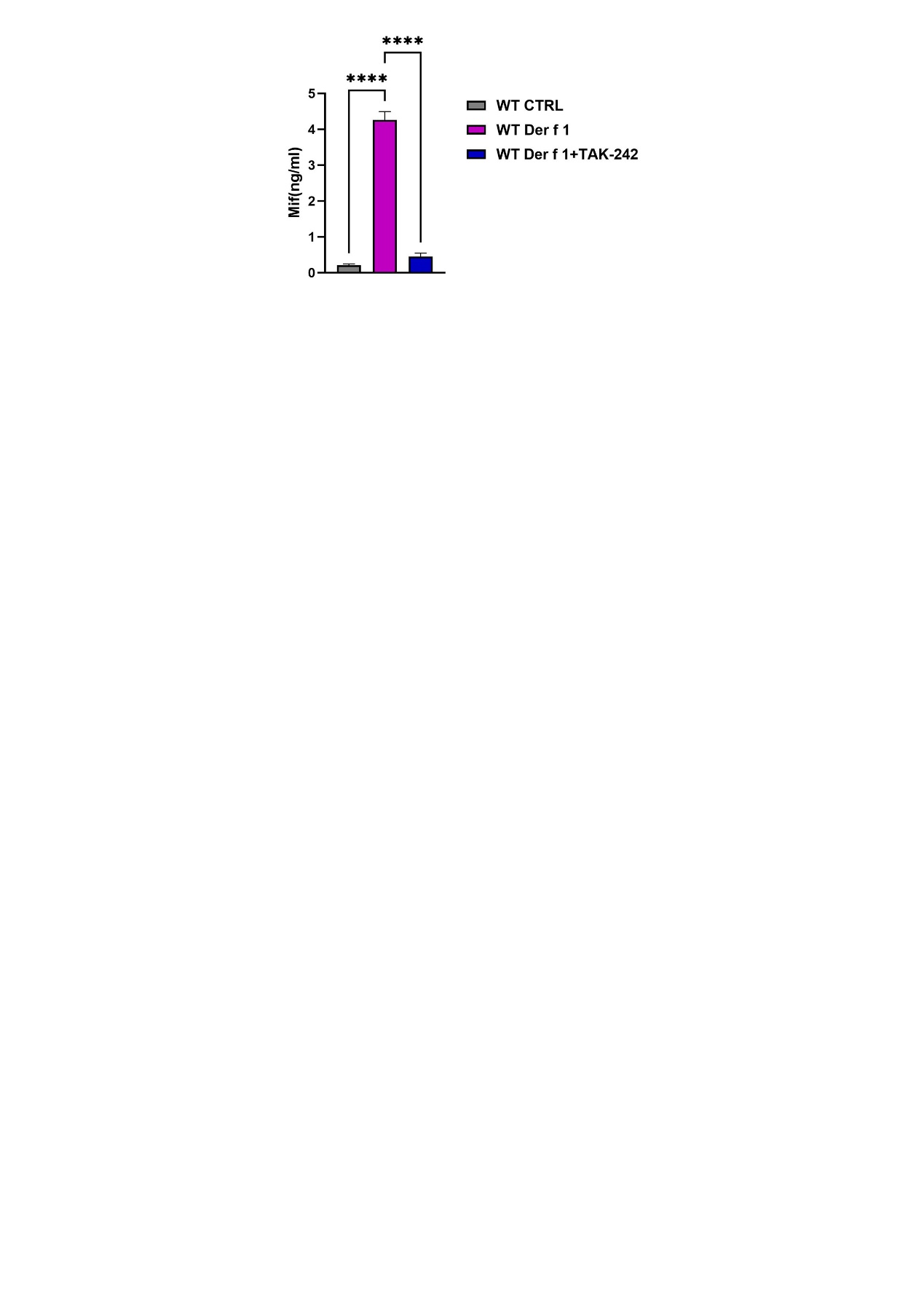


**Figure S6. MIF release in the supernatant of mouse primary keratinocytes treated with Der f1 or the TLR4 antagonist TAK-242.** ELISA of MIF showed that mouse primary keratinocytes (1.5*10^5^ cells) treated with *Der f1* (10μg/μl)+TAK-242 (10μM) for 12 hours inhibited the MIF release induced by *Der f1* stimulation. (1.5 × 10⁵ cells per group). One-way ANOVA. ****p < 0.0001. WT, wild type.


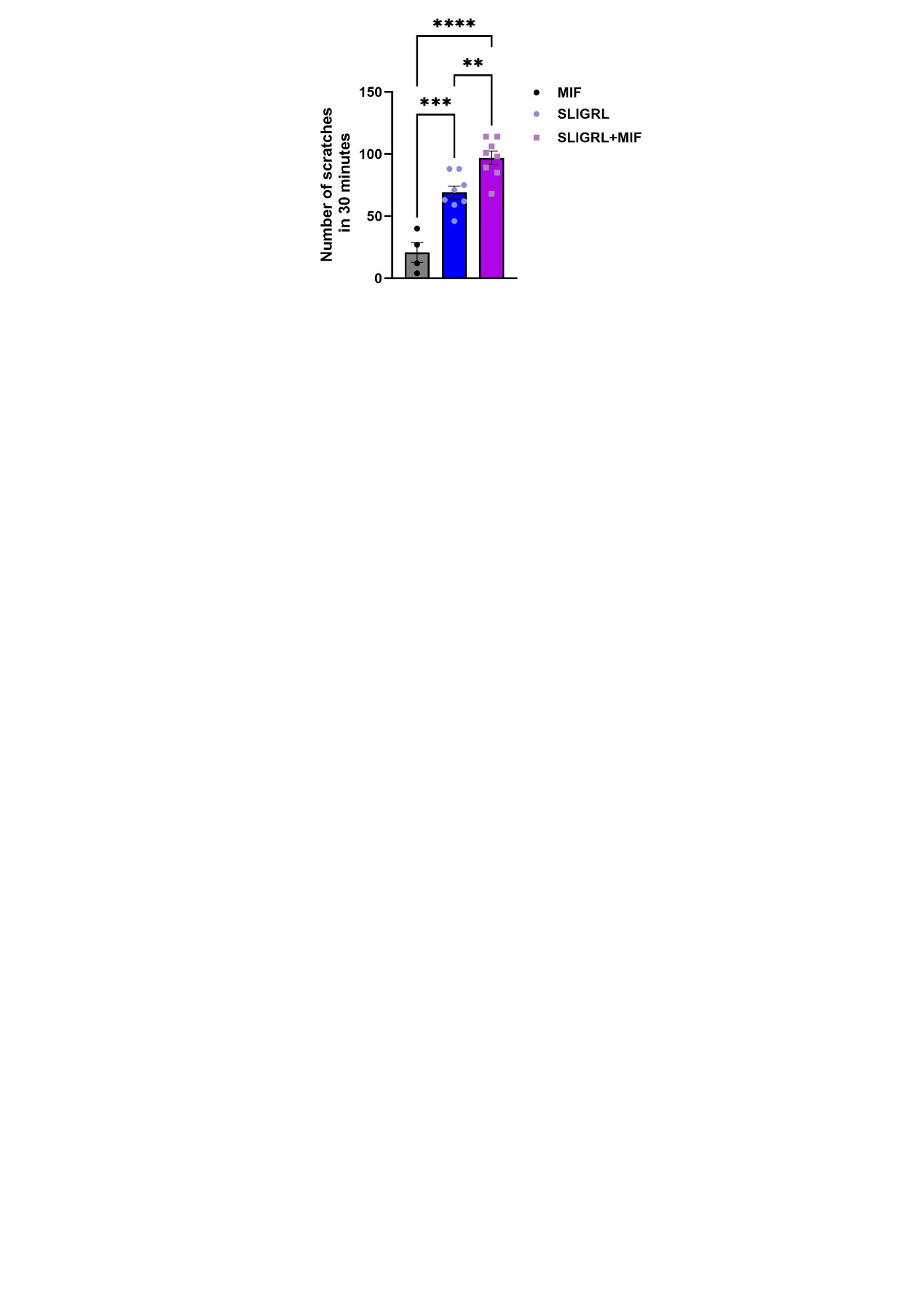


**Figure S7. MIF synergized with PAR2 agonist SLIGRL to promote mice acute itch.** Mice scratching bouts increased obviously induced by intradermal injection of SLIGRL (50μg) +MIF (0.5μg) than injecting SLIGRL only group. One-way ANOVA, n=4-8, ** p < 0.01, *** p < 0.001, ****p < 0.0001.

**Table S1. Primers for quantitative PCR.**

| mMif  Gene ID:17319 | Up | 5′-TTAGCGGCACGAACGATCC -3′ |
| --- | --- | --- |
|  | Down | 5′-ACAGCAGCTTACTGTAGTTGC -3′ |
| mKif13b  Gene ID:16554 | Up | 5′-GCTCTGTAGTGGACTCTTTGAAC -3’ |
|  | Down | 5′-TTTGGGGTCAAGAAGGTCTCG -3’ |
| mIL-4  Gene ID:16189 | Up | 5′-GGTCTCAACCCCCAGCTAGT -3’ |
|  | Down | 5′-GCCGATGATCTCTCTCAAGTGAT -3’ |
| mIL-13  Gene ID:16163 | Up | 5′-CCTGGCTCTTGCTTGCCTT -3’ |
|  | Down | 5′-GGTCTTGTGTGATGTTGCTCA -3’ |
| mTslp  Gene ID:53603 | Up | 5′-ACGGATGGGGCTAACTTACAA -3’ |
|  | Down | 5′-AGTCCTCGATTTGCTCGAACT -3’ |
| mArg1  Gene ID:11846 | Up | 5′-CTCCAAGCCAAAGTCCTTAGAG-3’ |
|  | Down | 5′-AGGAGCTGTCATTAGGGACATC-3’ |
| mIl1b  Gene ID:16176 | Up | 5′-TTCAGGCAGGCAGTATCACTC-3’ |
|  | Down | 5′-GAAGGTCCACGGGAAAGACAC-3’ |
| mIl6  Gene ID:16193 | Up | 5′-TAGTCCTTCCTACCCCAATTTCC-3’ |
|  | Down | 5′-TTGGTCCTTAGCCACTCCTTC-3’ |
| mIl10  Gene ID:16153 | Up | 5′-CTTACTGACTGGCATGAGGATCA-3’ |
|  | Down | 5′-GCAGCTCTAGGAGCATGTGG-3’ |
| mGapdh  Gene ID:14433 | Up | 5′- AGGTCGGTGTGAACGGATTTG -3′ |
|  | Down | 5′- TGTAGACCATGTAGTTGAGGTCA -3′ |

**Table S2. KC_cluster identity**

| **Cell type** | **Genes** |
| --- | --- |
| Undifferentiated KC | *KRT5, KRT14, CCL27A, CXCL14, KRT15* |
| Basal_1 | *TSLP, IGFBP3* |
| Basal_2 | *COL23A1, FST* |
| Basal_3 | *IL31RA, POSTN* |
| Differentiated KC | *KRT1, KRT10, KRTDAP* |
| Spinous_1 | *S100A9, S100A8* |
| Spinous_2 | *SKINT6, KRT6A* |
| Inner root sheath cell | *KRT71, KRT73, TCHH* |
| Proliferating KC | *MKI67, TOP2A, HIST1H2AP, HIST1H2AE* |

**KC：Keratinocytes.**
